# Chemsex: core knowledge for emergency medical service responders

**DOI:** 10.29045/14784726.2024.12.9.3.63

**Published:** 2024-12-01

**Authors:** Peter Kingsley

**Affiliations:** London Ambulance Service NHS Trust ORCID iD: https://orcid.org/0000-0002-4547-5179

**Keywords:** chemsex, emergency medical services, paramedic practice

## Abstract

**Aim:**

The aim of this professional practice article is to increase awareness and knowledge of chemsex among emergency medical service (EMS) clinicians.

**Background:**

EMS clinicians can expect to be called on to respond to medical emergencies across the range and breadth of human behaviours, some of which will take them into areas they are unfamiliar with and/or that involve illegal activity. It is likely that many EMS clinicians would regard chemsex as one such area. A secretive activity, largely occurring out of sight of wider society, chemsex involves the planned use of specific drugs to enhance, prolong and sustain sexual experiences. Most chemsex is consensual, with participants engaging in it because they derive pleasure and enjoyment from the activity. Many do not regard their participation as problematic and so are highly unlikely to have previously discussed this aspect of their lives with a medical or harm-reduction professional. Engagement in chemsex does, however, carry significant risks of both mental and physical harms. When something goes wrong at an event, EMS can expect to be called to respond.

In this article, chemsex scenarios are combined with literature drawn from a range of sources to explore multiple aspects of chemsex from the perspective of EMS clinicians.

**Conclusion:**

Chemsex invokes a complex interaction between physical health, mental health, social care, addiction medicine, sexual health and criminal justice. In providing a community-based response, EMS clinicians are uniquely placed as the only element of healthcare that sees chemsex participants at the event location, often while the incident is still going on. Equipping responders with core knowledge of chemsex activities will ensure they are best able to provide a response that is knowledgeable, patient-centred and offers unconditional positive regard. Clinicians that are chemsex-aware will be in a better position to recognise and understand the drugs that may have been taken and their associated toxidrome and appreciate the significant risk of physical and mental trauma. They will also recognise they are in a unique and privileged position and feel confident to engage in harm reduction with this very high-risk and largely unseen cohort of patients.

## Introduction

Public trust in emergency medical services (EMS) clinicians stems from the understanding that emergency responders are professional and knowledgeable, interacting with all sections of society without prejudice or judgement. Such a privileged, trusted and, in many respects, unique position comes with the responsibility to make every clinical contact count by dealing with people with care, compassion, empathy and positive regard.

To maintain this laudable standard, individual EMS clinicians must maintain an awareness of activities that are prevalent in the community they serve; some of these activities may be illegal and hidden from wider view and may involve participation by sub-cultures or groups. One such activity is ‘chemsex’.

In this article, the author will draw on over 20 years of experience and expertise as an EMS clinician working in a large UK city. During this time, he has responded to many incidents where chemsex activity has gone wrong. Using (fictional) scenarios based on these experiences, he provides an insight into the challenges presented to EMS when participation in chemsex goes awry and requires medical intervention. Personal reflections are combined with evidence from literature identified through searches utilising Google and Google Scholar and drawn from a wide range of sources, including peer-reviewed journals, online content from health advocacy groups and mainstream and specialist media outlets.

It is hoped that this article will enable EMS practitioners to better identify an incident that is potentially related to chemsex and, therefore, be in a better position to offer appropriate assistance, as well as compassion and understanding.

## What is chemsex?

The term ‘chemsex’ emerged in the late 1990s to describe a specific sub-type of sexualised drug use: a sub-culture in which certain drugs, behaviours and language are integral to the activity (Stuart, 2019). A socially constructed concept, chemsex does not have a fixed set of defining characteristics or criteria; however, certain elements are common to many who engage in it. Maxwell et al. (2019, p. 74) described chemsex behaviours as ‘the use of specific drugs before or during planned sex to facilitate, initiate, prolong, sustain, and intensify the encounter’. Chemsex has been the subject of multiple research studies and has featured in news articles and documentaries across a range of niche and mainstream media channels (MEN R US, 2024). Recent media articles with an EMS focus include the number of chemsex-related incidents EMS attend (Lee, 2024), the interaction between EMS, chemsex participants and the police (Wilkinson, 2024) and EMS involvement in chemsex harm reduction (Strudwick, 2023). Public Health England (2015) has identified chemsex as an issue of concern.

## Who and where?

Chemsex is an activity predominantly, but not exclusively, practised by a relatively small subgroup of gay, bisexual and other men who have sex with men (MSM) (Bourne et al., 2014). More recent drug trend analysis identified a similar predominantly MSM demographic, but also found increasing participation among those who identify as transgender (Ralphs et al., 2023).

Chemsex involves a range of activities in varied settings. How an individual chooses to participate will depend on factors including their own personal preference and practical considerations, such as what is available locally (Ahmed et al., 2016; Maxwell et al., 2019; M⊘ller, 2020). Examples of chemsex activity include:

- A group of men arranging to meet (‘hook up’, ‘party and play’) via geosocial apps at a private address to engage in sex with multiple partners over many hours/days.
- A monogamous couple using drugs as part of their sex lives.
- Activity taking place at licenced sex-on-premises venues (e.g. sauna, fetish nightclub).
- A participant, alone in their own home, using drugs while engaging in sexual activity with others via online video chat rooms (‘cybersex’).
- A sex worker taking drugs before meeting with a client.

While sex-on-premises venues still exist, there has been a steady decline in their number in recent years, a change driven by factors such as gentrification, rising business costs and falling attendances (as the MSM scene has increasingly moved online). This decline accelerated as many businesses did not survive the COVID-19 lockdowns (Day, 2022). From an EMS perspective, sex-on-premises locations present safer spaces for chemsex, as they employ sober staff who can be trained in harm reduction and first aid. Such venues also present harm-reduction organisations with an opportunity to engage with participants (Bourne et al., 2014). A consequence of the decline in premises has been to move chemsex activity into private spaces, hidden from wider society (Wilkinson, 2024).

COVID-19 lockdowns may also be associated with an increase in chemsex activity. An online survey of sexual health service users in London identified 157 people who participated in chemsex during the lockdowns. Of these, 26% (42/157) engaged in more chemsex than usual and 6.4% (10/157) tried chemsex for the first time during this period, citing isolation, anxiety, loneliness and boredom as reasons (Hyndman et al., 2021). There were similar results from a survey of sexual health service users in Brighton (Ringshall et al., 2022). A reported widespread perception within the gay community in Manchester of a significant increase in chemsex post the lockdowns ending may reflect the legacy of this increased activity (Ralphs et al., 2023).

Based on a keyword search of dispatch call logs, the London Ambulance Service NHS Trust (2023) estimated that they dealt with an average of one chemsex-related incident every day between January 2021 and September 2022.

## Chemsex drugs

The three drugs (‘chems’) most closely associated with the current UK chemsex scene are crystal methamphetamine, gamma-hydroxybutyrate/gamma-butyrolactone (GHB/GBL) and mephedrone (Hibbert et al., 2021; Ralphs et al., 2023; Stuart, 2019). These have emerged as the preferred drugs specifically because they provide the user with the desired effects to enable chemsex (mental disinhibition, enhanced empathy and social connection and sustained physical stamina). Other drugs, including ketamine, inhaled nitrates and erectile dysfunction medications, may be present at a chemsex event; however, these are most frequently taken in addition to the ‘chems’. Table 1 summarises key characteristics of these agents, the desired effects, findings on clinical examination and assessment and features of severe/life-threatening toxicity. 

**Table 1.** Summary of the drugs most frequently encountered in the UK chemsex scene, desired effects in the chemsex context, findings on clinical examination and assessment and features of severe/life-threatening toxicity.

Drug	Type of drug	Typical form	Desired effects (why it is used in chemsex)	Toxidrome/findings on examination	Severe/life-threatening toxicity
Crystal methamphetamine	Amphetamine Potent psychostimulant Increases dopamine, noradrenaline and serotonin in the brain	Crystalline rocks	Euphoria Heightened awareness and mental arousal Heightened emotion Enhanced self-esteem Increased energy Capacity to stay awake for many hours/days Shortened refractory time between sexual acts	Sweating Dilated pupils Tremor Confusion Anxiety Headache Abdominal pain Hallucinations Paranoia Palpitations Hypertension Hypoglycaemia Chest pain Narrow complex tachycardia	Hyperthermia (particularly if >39°C) Coma Seizures/convulsions Myocardial infarction Pulmonary oedema Rhabdomyolysis Severe metabolic acidosis Ventricular tachycardia leading to ventricular fibrillation Multi-organ failure
GHB (gamma-hydroxybutyrate) GBL (gamma-butyrolactone)	Sedative Naturally occurring neurotransmitter in the brain GHB is manufactured by processing its precursor, GBL Both GHB and GBL can be taken and are popular on the chemsex scene When taken, GBL is metabolised to GHB	White salt (GHB) Clear liquid (GBL)	Increased confidence Disinhibition Widespread physical and mental relaxation Increased sexual arousal	Low-dose effects similar to alcohol Anaesthetic at higher doses Hypersalivation Ataxia Miosis Impaired judgement/coordination/reaction time Slowed/irregular/shallow breathing Vomiting Blackouts/amnesia Unconsciousness (‘G-sleep’) Cyclical episodes of reduced level of consciousness interspersed with periods of agitation	Ataxia Severe respiratory depression Seizures Coma Loss of airway reflexes ECG abnormalities including AV block and prolongation of cardiac cycle Hypotension Severe metabolic acidosis
Mephedrone	A psychoactive stimulant Synthetic cathinonine Chemically related to amphetamines – acts on the same neuronal pathways but less potent	Oral tablet White powder	Empathomimetic Enhanced feelings of empathy, disinhibition, social acceptance, connection with others Heightened sexual arousal	Similar to crystal methamphetamine	Severe toxicity typically seen when taken in combination with other serotonergic drugs (e.g. amphetamines) Can lead to serotonin syndrome, with symptoms including confusion, agitation, sweating, increased heart rate and muscle spasms
Ketamine	Dissociative anaesthetic Potent antagonist at NMDA receptors Mild μ-opioid receptor agonist Mild α1 and β2 adrenoceptor agonist	Clear liquid White or off-white powder Oral tablet	Enhanced feelings of empathy, disinhibition, social acceptance, connection with others Feeling detached from own body Lowered sensitivity to pain	Highly dose dependent Inebriated behaviour (similar to alcohol) Auditory and visual hallucinations Agitation/anxiety/panic Ataxia/confusion/clumsiness Nystagmus Miosis or mydriasis Slurred speech Blank staring Tremors Hyperreflexia Clonic movements Increased muscle tone Increased heart rate Increased blood pressure	Loss of consciousness (‘k-hole’) Catatonic-like posturing Seizures Coma Rhabdomyolysis Acute kidney injury Acute lung injury Hyperthermia Respiratory depression and arrest (particularly if taken with depressant drug, e.g. GHB/GBL)
Avanafil Sildenafil Verdenafil Tadalafil Alprostadil	Oral phosphodiesterase type-5 inhibitor Alprostadil – synthetic prostaglandin causing vasodilation local to site of administration Intended for use in treatment of erectile dysfunction	Oral tablet Alprostadil – injection, suppository or topical gel	Increased male erection Reduced refractory time between sexual acts	Vasodilation Hypotension Headache Dizziness Flushing GI upset Priapism	Potentially significant vasodilation if taken with nitrates (see ‘poppers’ below) Prolonged priapism is medical emergency Taken with stimulant drugs may initiate hypertensive crisis
Amyl nitrate Butyl nitrate Isobutyl nitrate Isopropyl nitrate	Nitrate Inhaled vasodilator Available widely and legally sold as ‘room odouriser’	Aromatic liquid contained in a small glass bottle	Aphrodisiac Relaxes smooth muscle	GI upset Blurred vision Sweating Throat irritation Contact dermatitis Headache Dizziness	Hypotension – profound if taken with other vasoactive drugs Methaemoglobinaemia (may be severe and life threatening if drug is ingested)

Data sourced from Joint Formulary Committee (2024) and National Poisons Information Service (2024).

### Identifying which drug may have been taken

Identifying a particular drug from clinical findings alone can be difficult and unreliable, particularly in the context of co-ingestion of agents (Hockenhull et al., 2016; Nutbeam et al., 2023). In most situations, the patient or someone else at the event will tell the attending EMS crew what drugs may have been taken. If required, further information can be gained from a visual examination of items in the immediate vicinity of the patient. Table 2 details some slang words related to the chemsex scene, methods of drug administration and associated paraphernalia that EMS may encounter when dealing with a chemsex incident.

**Table 2.** Chemsex-related slang words, methods of drug administration and associated paraphernalia.

Drug (‘chems’) slang words	Appearance/method of administrating	Evidence paraphernalia
Crystal methamphetamine: ‘tina’ ‘meth’ ‘ice’ ‘T’, e.g. ‘parTy and play’	Smoked by burning in a glass pipe (‘meth pipe’) Ground to a powder and snorted or mixed with water and injected intravenously (‘slamming’) Administered rectally (‘booty bump’)	Small plastic bag containing fragments of crystals (similar in appearance to ground glass) Small glass pipe with a soot/heat-stained bowl on one end (‘meth pipe’) indicates inhaled crystal methamphetamine Venous tourniquet, 1–3-ml syringe with needle attached, soot/heat-stained spoon or needle injection marks on the patient’s skin (‘track marks’) indicate intravenous administration (‘slamming’)
GHB (gamma-hydroxybutyrate)/GBL (gamma-butyrolactone): ‘G’ ‘liquid ecstasy’	A white salt (GHB) or a clear liquid (GBL) Salt is dissolved in a small quantity of water to form a clear, colourless and tasteless liquid Typical doses: 0.5–3 ml every 2–3 hours Onset 15–20 minutes Taken orally (most common), injected or administered rectally (using e.g. a tampon)	Small plastic bag containing white salt-like powder 10–20-ml bottle containing a clear liquid 3–5-ml syringe without a needle, pipette or small plastic squeezable bottle (e.g. the fish-shaped soy sauce container from take-away sushi meal) suggests oral dosing Hand-written chart (G-chart) or countdown timer running on a mobile phone – used to monitor dosing/reduce risk of overdose Venous tourniquet, 1–3-ml syringe with needle attached or needle injection marks on the patient’s skin (‘track marks’) indicate intravenous administration
Mephedrone: ‘meph’ ‘mcat’ ‘drone’ ‘meow meow’	In powder form can be snorted, mixed with water and injected or administered rectally (‘booty bump’) Tablets taken orally	Small plastic bag containing white powder Traces of white powder on a smooth surface suggest nasal snorting Venous tourniquet, 1–3-ml syringe with needle attached or needle injection marks on the patient’s skin (‘track marks’) indicate intravenous administration
Ketamine: ‘K’ ‘special K’ ‘ket’	In liquid form can be injected Powder can be snorted	Small plastic bag containing white powder Traces of white powder on a smooth surface suggest nasal snorting Venous tourniquet, 1–3-ml syringe with needle attached or needle injection marks on the patient’s skin (‘track marks’) indicate intravenous administration
Avanafil Sildenafil Verdenafil Tadalafil Alprostadil	Oral tablet Alprostadil: Injection – supplied as kit with powder vial and syringe pre-filled with water for injection. Mixed prior to administration and injected directly into penis Suppository – inserted directly into urethra Topical gel – applied directly to penis	Discarded tablets Empty packets Discarded syringe Bruising at site of injection if administered incorrectly Localised irritation if suppository used
Amyl nitrate/butyl nitrate/isobutyl nitrate/isopropyl nitrate: ‘poppers’	Effect is achieved through inhaling vapours	Small brown glass bottle, typically labelled, e.g. ‘room odouriser’ Contact dermatitis around the nostrils can indicate regular use

Information sourced from MEN R US (2024) and Department for Health and Social Care (2024).

## Recognising chemsex

Scenario 1 illustrates the approach adopted by the attending EMS clinicians to enable the patient to feel comfortable revealing that they had been at a chemsex event.

### Scenario 1

EMS responded to a report of a distressed person sitting on the pavement, alone, in the middle of the night. On arrival they found the patient was awake and talking. Employing an open, honest and non-judgemental approach, they gained his trust. The patient told the crew he had been at a party organised through a hook-up app, where he took GHB and mephedrone. He stated he thought he had passed out at some point. He remembered waking with the party continuing around him. He left, still intoxicated, and walked around the local area, lost and disorientated. The crew recognised that the effects of the drugs were rapidly wearing off and were satisfied that the patient was now able to make his own decisions. He declined all offers of assistance and arranged for a mini-cab to get himself home.

Clearly, a naked or semi-clothed person collapsed on the floor of a bedroom, hotel room, sauna or fetish nightclub, with drug and sex paraphernalia around them, would make it easier to identify the situation as chemsex. A more challenging scenario would be one in which a participant at an event has become distressed (e.g. due to drug intoxication) and has walked away alone. They may then be found some distance away, collapsed or in a highly agitated / psychotic state. Such calls may originate as a ‘collapse ? cause’ dispatch. In the absence of any other clues, it may be impossible to determine if the incident is related to chemsex.

## Chemsex harms

A systematic review of qualitative studies (Maxwell et al., 2019) identified that many chemsex participants do not regard their engagement as problematic and feel in control of their drug use. Over 50% of regular chemsex participants in one study reported never having sought professional help for drug use (Bourne et al., 2014). However, the same study also reported many more participants who described mental and physical ill health resulting from their participation in chemsex. 

Identified harms range from physical and mental health to issues with ‘lost time’, employment and finance, personal relationships and harm to the wider gay community (Bourne et al., 2014). Scenarios 2 and 3 illustrate that engaging in chemsex comes with significant risks. Disinhibition through drug intoxication can lead to accidental overdose, contracting a sexually transmitted disease or blood-borne virus from unintended unprotected sex or physical injuries resulting from, for example, rough penetrative sex. Drug-induced paranoia, agitation or psychosis can result in someone sustaining serious trauma injuries; for example, from running into fast-moving traffic or falling from height (Carthy et al., 2021; Maxwell et al., 2019; Nutbeam et al., 2023).

### Scenario 2

An EMS crew are treating an unconscious male in the bedroom of a private house. Others present tell them the patient has taken GHB and, as the drug took effect, he became ‘happy, high and horny’. In his intoxicated state he drank the contents of a shot glass, thinking it was water ‒ it was GBL. A few minutes later he ‘fell asleep’. Others at the party watched over him, only calling EMS when his breathing slowed and he started snoring. Clinical assessment confirmed a deeply sedated patient. Knowing the GHB/GBL toxidrome, the EMS crew prepare equipment in anticipation of the patient requiring airway and ventilation support and establish IV access in readiness for seizures.

### Scenario 3

Police have responded to reports of an adult male in the street shouting, screaming and running in and out of moving traffic, and have requested urgent EMS. On arrival the EMS crew find the patient is handcuffed and being physically restrained on the ground by five officers. The patient is highly agitated, with seemingly relentless violent physical activity, hot to the touch and sweating profusely. Police officers show the EMS crew the contents of the patient’s backpack, which include a sex toy, a small glass pipe and a bag containing traces of crystalline powder. Based on their clinical assessment and the drug paraphernalia, the crew identify that the patient has most likely taken crystal methamphetamine. This has precipitated a severe acute behavioural disturbance. Working with the police, they optimise the physical restraint and request urgent specialist critical care support. The patient is subsequently chemically sedated/tranquilised and conveyed to the local emergency department. He is later transferred to the intensive care unit.

Chemsex drugs have the potential to be highly addictive and some participants go on to develop physical and mental dependency on one or more of them. What began as chemsex months or years previously becomes only about the ‘chems’ (Moreno-Gámez et al., 2022). As illustrated in Scenario 4, people may seek emergency medical help when they are in an acute mental and/or physical health crisis resulting from, for example, acute withdrawal. Regular users can develop a tolerance to the drugs, resulting in them taking larger doses to provide the same effect (Carthy et al., 2021; Maxwell et al., 2019). Acute withdrawal can result in intense cravings for the drug(s), anxiety, agitation and confusion. This may rapidly escalate to hallucinations, delirium and seizures (Hibbert et al., 2021; Moreno-Gámez et al., 2022).

### Scenario 4

An EMS crew are assessing a 45-year-old patient presenting with extreme anxiety and chest pain. It is the fourth such call to this patient in the past 24 hours. He has an established pattern of calling EMS, attending the emergency department, self-discharging and then calling EMS again a few hours later. He describes long-term problem drug use associated with crystal methamphetamine dependency. He says he first took the drug at a chemsex hook-up over 20 years ago.

A retrospective review of attendance data from two Central London hospitals between 2005 and 2018 identified 1244 incidents of patients presenting with methamphetamine intoxication (Harnett et al., 2022). Results revealed a year-on-year increase in cases, rising from four in 2005 to 294 in 2018. Approximately 25% of patients had taken only methamphetamine, with the remainder having taken methamphetamine and one or more other agents (Table 3). The most frequent co-ingestant was GHB/GBL (‘G&T’). Although combining methamphetamine with GHB/GBL was associated with a four-fold increase in admission to a high-dependency or intensive care unit (2.5% lone methamphetamine versus 11.5% methamphetamine with GHB/GBL), overall in-hospital mortality was very low at 0.001% (1/1244) (Harnett et al., 2022).

**Table 3.** The most frequent co-ingestants with methamphetamine.

Methamphetamine co-used substances	
GHB/GBL^a^	461 (54.2%)
Ethanol	166 (19.5%)
Mephedrone	95 (11.1%)
Cocaine / crack cocaine	68 (8.0%)
MDMA/ecstasy	40 (4.7%)
Ketamine	34 (4.0%)
Diazepam	31 (3.6%)
Cannabis	26 (3.1%)
Sildenafil	26 (3.1%)
Heroin	17 (2.0%)

^a^GHB/GBL: gamma-hydroxybutyrate/gamma-butyrolactone

Source: Harnett et al. (2022). Reproduced with permission from the author.

A retrospective review of 6633 post-mortem blood samples analysed in a single central London laboratory between 2011 and 2015 found that 61% of samples contained GHB/GBL at levels high enough to have contributed to, or caused, the death (Hockenhull et al., 2016). In approximately two-thirds of cases, GHB/GBL was a co-ingestant with methamphetamine and/or mephedrone. The authors concluded that the number of deaths is disproportionally high considering the relatively low level of GHB/GBL use in society (Hockenhull et al., 2016). With no routine testing of post-mortem samples for GHB/GBL, technical challenges in accurately measuring levels of the drugs post mortem and the continued expansion of the chemsex scene since 2016, the true number of deaths per year is likely to be significantly higher than suggested by both studies (Hockenhull et al., 2016; Ralphs et al., 2023). Approximately 80% of the deaths in Hockenhull et al.’s (2016) study occurred in the community. It is likely that many (or all) of these were included in a review of 55 GHB/GBL-related deaths in London between June 2011 and October 2015. Forty-five of these deaths occurred in a private residence or hotel room, six in a sauna and only three in a public place (Metropolitan Police Service, 2019).

From an EMS perspective, the papers in this section demonstrate that ‘chems’ are toxic and taking them can be fatal. When deaths occur, they are highly likely to happen in the community, in private spaces, away from view. They also signal that if the patient arrives at hospital alive, they are highly likely to survive. 

## Why engage in chemsex?

Appreciating that there are multiple reasons why someone may choose to engage in chemsex is important if EMS clinicians are to succeed in being empathetic and understanding to participants. A thematic review of study participant interviews (Bourne et al., 2014) identified six predominant motivating factors:

- Facilitating sexual confidence
- Increasing sexual desire and libido
- Intimacy and sexual connection
- Sexual longevity and partner turnover
- Enabling sexual adventure
- Sexual (un)happiness and making sex better.

**Figure 1:**
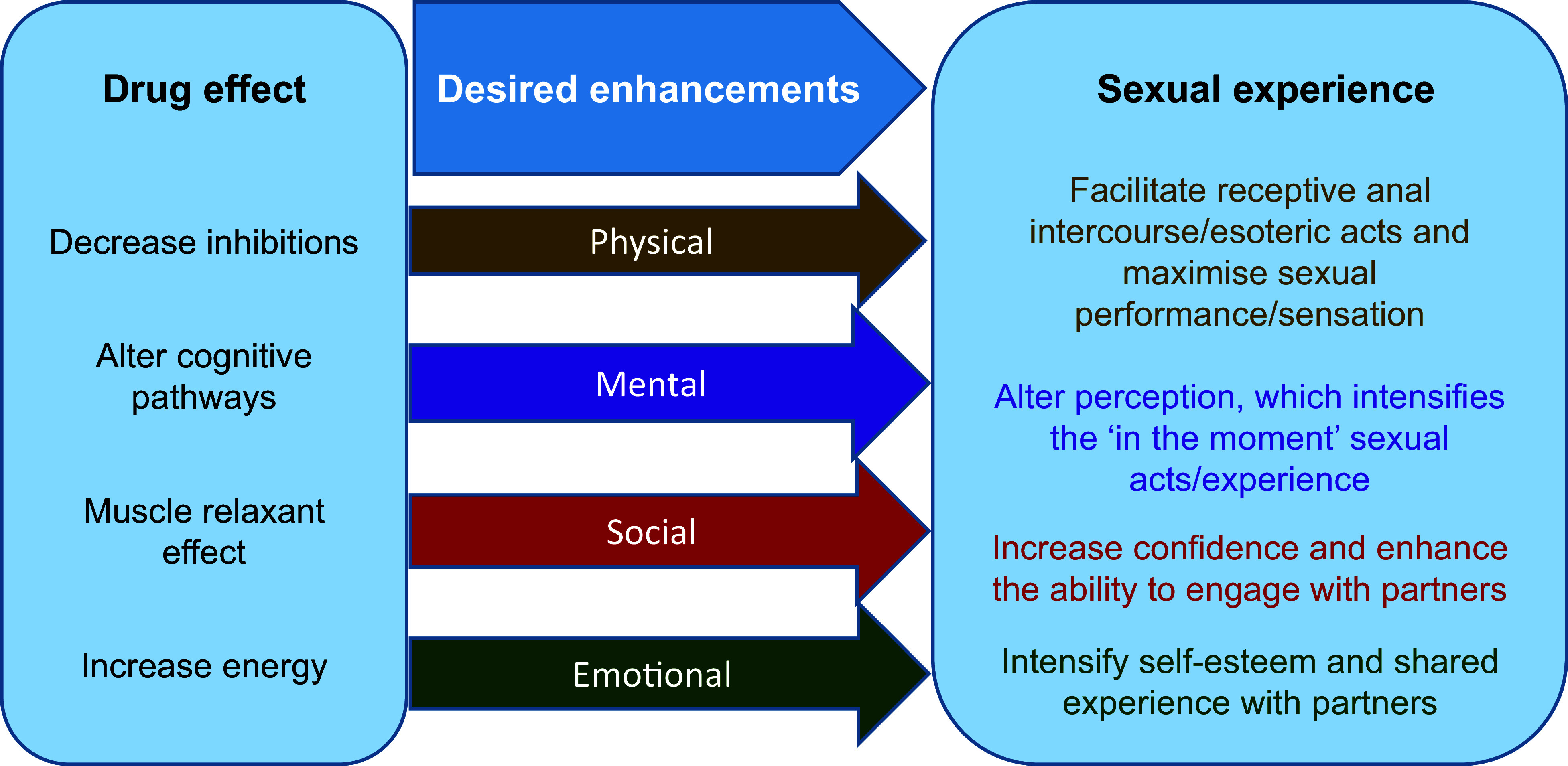
Figure 1. Expected drug effects on the sexual event.

Source: Maxwell et al. (2019). Reproduced with permission from the author.

In a systematic review, Maxwell et al. (2019) identified motivators across four broad domains (desired enhancements) and linked these with characteristics of the drugs (Figure 1).

## The justice context

The characteristics of the drugs (loss of consciousness, amnesia, disinhibition and loss of muscle tone) make them appealing to those with criminal intent. As a liquid, GHB/GBL can be added to drinks or lubricants (‘spiking’) without the recipient knowing. Rendered unconscious, victims can be subjected to sexual assault, robbery or burglary (Carthy et al., 2021; Home Office, 2021; McStravick, 2022).

In 2016, a man was sentenced to a whole-life term of imprisonment for four murders and 18 serious sexual assaults committed over an 18-month period in London. These offences were facilitated by the use of GHB and geosocial apps (Sandford, 2016). In Manchester, a serial rapist used the same drug to carry out over 150 rapes and sexual assaults against multiple men over a two-year period (Abbitt, 2021).

Such tragic and disturbing events are rare and do not define chemsex. More commonly, the unfortunate results of participation are death from accidental overdose, long-term physical and/or mental ill health and loss of livelihood resulting from losing a job or being unemployable. Issues around consent and mental capacity while intoxicated leave many participants experiencing unease and distress as they recount personal experiences (Bourne et al., 2014; Carthy et al., 2021; Marshall, 2023; Strudwick, 2018).

Many of the toxic effects of the drugs can be addressed if the patient receives early medical aid (Hockenhull et al., 2016; National Poisons Information Service, 2024). However, research has identified reluctance among chemsex participants to call the EMS, reflecting a belief that they will co-respond with the police. A lack of trust or confidence in the police, feelings of shame or stigma and a desire to keep their participation private result in a hesitancy to call for help when it is clearly needed (Freestone et al., 2023; MEN R US, 2022). This presents a challenge for EMS responders, who must balance the desire to help and support their patients, respecting their right to confidential treatment, against their knowledge that serious criminality exists within the chemsex context.

In situations such as an unexpected death or where there is a risk to personal safety, the police must attend (Nutbeam et al., 2023), but these are the exception rather than the norm. More commonly, determining if police should be informed requires the attending clinician to make a careful and considered assessment of the situation. Patients have a right to expect their personal information to remain confidential. Guidance issued by the UK General Medical Council (GMC) in 2018 stresses the benefits to the individual and wider society when people feel comfortable and confident in seeking advice and treatment. However, it cautions, ‘[. . .] there can be a public interest in disclosing information to protect individuals or society from risks of serious harm, such as from [. . .] serious crime’.

The guidance goes on to discuss consent:

*If it is not practicable or appropriate to seek consent, and in exceptional cases where a patient has refused consent, disclosing personal information may be justified in the public interest if failure to do so may expose others to a risk of death or serious harm. The benefits to an individual or to society of the disclosure must outweigh both the patient’s and the public interest in keeping the information confidential.*

*Such a situation might arise, for example, if a disclosure would be likely to be necessary for the prevention, detection or prosecution of serious crime, especially crimes against the person. When victims of violence refuse police assistance, disclosure may still be justified if others remain at risk [. . .].* (GMC, 2018, paragraphs 64 and 65)

Scenarios 5, 6 and 7 describe incidents in which the ethical, legal and moral obligations on EMS are balanced (College of Policing, 2023; GMC, 2018).

### Scenario 5

Two men ‘hook up’ via a smartphone app. They both take GHB. One of them collapses and becomes unconscious and EMS are called. On arrival the crew find a calm scene, with nothing to indicate any non-consensual activity has taken place. The crew recognise that in this situation there is no ‘serious crime’ and so no indication to involve the police. Understanding that this is a chemsex event, they focus on providing excellent clinical care in a manner that is compassionate, understanding and non-judgemental. They treat the patient’s personal information as confidential.

### Scenario 6

EMS are called to a hotel where staff have found an unconscious man alone in a bedroom. Sex and drug paraphernalia indicate this is likely to have been a chemsex event. The patient is unconscious and breathing slowly. He is naked and they can see early signs of bruising on his arms. Recognising likely methamphetamine and GHB/GBL intoxication, the crew prioritise airway and breathing support, establish intravenous access in anticipation of seizures and expedite removal to hospital.

The EMS clinicians do not know what has happened; however, they are concerned the patient may have been the victim of a serious sexual assault and so they request the police to attend, and they inform the officer of their concerns. Aware the patient has not consented to having his personal information shared, the crew maintains confidentiality. They do tell the officer which hospital the patient is being conveyed to. On arrival, the crew inform the triage nurse of their concerns and the actions they have taken.

The crew complete a comprehensive set of notes detailing what they found, their concerns and the actions they took.

Hospital staff speak to the patient when he has recovered sufficiently to talk.

### Scenario 7

EMS receive a call from a distressed man. He tells the attending crew he was at a ‘hook-up’, where he took GBL and passed out. He thinks that while he was unconscious, he was sexually assaulted. He has injuries consistent with an assault that require hospital treatment. He does not want the police involved.

The crew recognise that this patient retains mental capacity and so it is for him to decide how he would like to deal with the situation. They respect his decision and maintain confidentiality.

Understanding the sensitivities and complexities of chemsex, they are able to engage with their patient and he agrees to go with them for further treatment.

The patient then reveals that when he left the event, there was another man unconscious on the floor. He thinks this man was also assaulted.

The crew recognise that this changes the situation. Regardless of what their patient wants, the concern now is that others may be at risk of serious harm. They act immediately and request the police to attend. They inform the attending officer of their concerns. They continue to maintain patient confidentiality and do not share any personal information without consent.

Prior to clearing from the call, they complete a comprehensive set of notes detailing their actions.

## Conclusion

Chemsex is a secretive activity, largely hidden from wider society, and participants typically attend an event willingly and with full knowledge of what is involved. Motivators to engage in chemsex are personal and unique to each individual and not all participants consider it problematic. Some may take part simply because they enjoy it; for others, participation reflects emotional and mental health disease and distress.

With activities ranging from multiple attendees at a private house, through to events hosted via online media, chemsex activity is not restricted by location. It follows that anyone working in EMS, regardless of where they work, may find themselves responding to an incident. It is important to recognise that EMS staff are uniquely placed as the only element of healthcare that sees chemsex participants in the event context.

Chemsex invokes a complex interaction between physical, mental and sexual health, social care, addiction medicine and criminal justice. Consequently, responding to incidents such as those described in this text can present complex challenges for EMS clinicians.

In order to provide non-judgemental, caring, compassionate and patient-centred healthcare, EMS staff should be able to:

- Recognise when an event may relate to chemsex.
- Understand why someone may choose to participate.
- Understand the drugs used and their associated risks.
- Anticipate clinical deterioration.
- Remain vigilant to the potential for significant physical injury.
- Remain vigilant to the potential for serious criminality.
- Understand the particular sensitivities around working with the police.
- Understand the legal, ethical and moral components to maintaining patient confidentiality.

When something goes wrong at an event and EMS attend, it will likely have a sobering effect on all those present. This represents a ‘reachable moment’. All of the scenarios included in this article are inspired by/reflective of real incidents attended by EMS clinicians. Each of them involved individuals who may not have previously engaged with healthcare providers in a chemsex context. In all of them, EMS has an opportunity to make a positive difference. For example:

- In Scenario 1 there is an opportunity to signpost harm-reduction advice before the patient leaves in the mini-cab.
- In Scenario 2 the other participants are watching EMS deliver airway and breathing support to someone who only an hour earlier was enjoying the same activity as them. This is sobering ‒ a ‘reachable moment’. Would they be receptive to some first aid advice so they can better manage a similar situation in the future? Or information on how to make GHB/GBL dosing safer?
- In Scenario 7 the patient has been the victim of a serious sexual assault. Why he does not want to involve the authorities is personal to him ‒ shame, stigma, embarrassment, a desire for privacy or a strong distrust of the police are all possibilities. EMS responders should be equipped to signpost him to organisations that offer support to victims of sexual assault.

Further research is needed to identify how prevalent chemsex is in the UK and its impact on EMS providers. Although the overall numbers of people involved in chemsex activities represent a very small sub-group of society, there are signs that the numbers are increasing. In addition to providing the clinical care required, EMS clinicians should actively seek to optimise any opportunity to promote health and harm-reduction interventions in this high-risk and difficult-to-reach group.

## Acknowledgements

The author gratefully acknowledges the support provided by Dr Ann Thornton in editing, proof-reading and formatting the final article. This article forms part of a much larger chemsex harm-reduction initiative being undertaken by the London Ambulance Service NHS Trust. This work is a collaboration between the author and colleagues Carly Lynch (Consultant Nurse for Mental Health) and Daniel Phillips (co-chair LAS LGBTQ+ staff network and Mental Health Paramedic Lead), together with Patriic Gayle from the Gay Men’s Health Collective. The author is grateful for their invaluable support and insight into chemsex. 

## Approvals

The author has received approval from the London Ambulance Service NHS Trust to publish this article.

## Conflict of interest

None declared.

## Funding

None.
